# Screening human cell lines for viral infections applying RNA-Seq data analysis

**DOI:** 10.1371/journal.pone.0210404

**Published:** 2019-01-10

**Authors:** Cord C. Uphoff, Claudia Pommerenke, Sabine A. Denkmann, Hans G. Drexler

**Affiliations:** Department of Human and Animal Cell Lines, Leibniz Institute DSMZ—German Collection of Microorganisms and Cell Cultures, Braunschweig, Germany; Centers for Disease Control and Prevention, UNITED STATES

## Abstract

Monitoring viral infections of cell cultures is largely neglected although the viruses may have an impact on the physiology of cells and may constitute a biohazard regarding laboratory safety and safety of bioactive agents produced by cell cultures. PCR, immunological assays, and enzyme activity tests represent common methods to detect virus infections. We have screened more than 300 Cancer Cell Line Encyclopedia RNA sequencing and 60 whole exome sequencing human cell lines data sets for specific viral sequences and general viral nucleotide and protein sequence assessment applying the Taxonomer bioinformatics tool developed by IDbyDNA. The results were compared with our previous findings from virus specific PCR analyses. Both, the results obtained from the direct alignment method and the Taxonomer alignment method revealed a complete concordance with the PCR results: twenty cell lines were found to be infected with five virus species. Taxonomer further uncovered a bovine polyomavirus infection in the breast cancer cell line SK-BR-3 most likely introduced by contaminated fetal bovine serum. RNA-Seq data sets were more sensitive for virus detection although a significant proportion of cell lines revealed low numbers of virus specific alignments attributable to low level nucleotide contamination during RNA preparation or sequencing procedure. Low quality reads leading to Taxonomer false positive results can be eliminated by trimming the sequence data before analysis. One further important result is that no viruses were detected that had never been shown to occur in cell cultures. The results prove that the currently applied testing of cell cultures is adequate for the detection of contamination and for the risk assessment of cell cultures. The results emphasize that next generation sequencing is an efficient tool to determine the viral infection status of human cells.

## Introduction

Although most bacterial (particularly mycoplasma), fungal and cross contamination (mix-up of different cell lines) of cell cultures can be detected conveniently with high sensitivity and specificity, virus infections still represent a challenge regarding their detection, evaluation and handling in cell culture technology and particularly in pharmacological and medical applications [1]. Accurate determination is impeded by structural heterogeneity of virus particles and their diverse life cycles in eukaryotic cells and higher organisms. The lack of knowledge of which viruses do possess the potential to infect different cultured cells and, in particular, which viruses are able to reproduce within the cells are further difficulties in this matter. Thus, until now there is no general and practical method for a comprehensive detection of viruses in cell cultures (which is, of course, similarly true for patients suffering from unspecified diseases). Usually, cell culture viruses (1) originate from an infection of a patient or donor, (2) are deliberately introduced into the cell culture (e.g. for immortalization), (3) might be transmitted secondarily during cell culture manipulation, e.g. xenotransplantation for tumorigenicity testing, by cross contamination from an infected culture, (4) by contaminated cell culture media supplements (e.g. fetal bovine serum; FBS) [2], or (5) from laboratory staff (e.g. adenovirus) due to poor aseptic practice or failure of microbiological safety cabinets.

In contrast to bacterial contaminations, most viral infections are species and tissue specific. However, some viruses bind to more or less ubiquitously expressed surface proteins of eukaryotic cells, for example poly- and xenotropic murine leukemia viruses (P-/X-MLV) [3] and bovine viral diarrhea virus (BVDV) [4]. Viral infections can be either productive, leading to the release of active viruses, or latent with no virus production. Latent infections can sometimes be triggered to evolve to a productive or lytic phase by various inducers during cell culture. If all cells are infected, virus infections cannot be eliminated from a cell culture.

An infection can pose a significant risk for patients when medical or pharmaceutical products are prepared using infected cell lines, but also for the user of infected cell cultures in a laboratory. For example, viral sequences, but fortunately no active viruses, were found in the interferon-alpha preparations produced with the Burkitt lymphoma cell line NAMALWA which is contaminated with squirrel monkey retrovirus (SMRV) [5, 6]. Furthermore, it is also likely that the viruses have an effect on the cell in an experimental setting. Thus, it is of utmost importance to know which kind of virus is present in the cells. If previously undetected viruses are found in cell lines, they also might indicate a possible link to carcinogenesis as was shown for hepatitis B virus (HBV), human T-lymphotropic virus (HTLV-1/-2), Epstein-Barr virus (EBV), some human papillomaviruses (HPV), and a few other viruses [7]. Several years ago, xenotropic murine leukemia virus related virus (XMRV) caused some sensation when it was found in a prostate carcinoma cell line and was subsequently erroneously linked to the tumor type and also to chronic fatigue syndrome [8]. Finally, the detection of human or non-human viruses in different cell lines would indicate the dissemination of viral contaminants via inappropriate cell culture practice as documented for murine leukemia viruses (MLV) [9, 10].

To perform risk assessment and for the characterization of cell lines present in the bank of the Leibniz Institute DSMZ–German Collection of Microorganisms and Cell Cultures (Braunschweig, Germany), we have tested all human and non-human primate cell lines for various human pathogenic viruses as well as for other non-human viruses possibly occurring in cell cultures. Although the risk that other human pathogenic viruses are present is extremely low, we have never been certain that any given cell culture is free of pathogens other than human or animal viruses that have been tested for by PCR. Next generation sequencing (NGS) and the new bioinformatics tools promise to be useful for the detection of already known and potentially new virus infections of cell cultures.

It had been shown that NGS does not only amplify the transcripts that map to the human genome in the case of RNA-Seq methods, but produces also reads in a significant portion that do not map to the human genome [11]. The same is true for sequences of whole exome (WES) and whole genome sequencing (WGS) methods. Concerning WES 40–60% of the resulting reads are off-target reads originating from unspecific binding of the primers, contamination of the primary material itself (upstream contamination), or contamination during the preparation or sequencing of the samples (downstream contamination) [12].

The development of free and publicly available interactive metagenomics analysis software and the availability of RNA-Seq, WES, and WGS data enabled us to screen cell lines for a multitude of viruses by screening of nucleotide sequences not mapping to the human genome. The results were compared and cross-evaluated with data based on PCR analyses. In this study, we first investigated the NGS data sets of cell lines already tested by conventional PCR for the same set of viruses by aligning all reads of a data set to the genome sequences of the individual virus genomes. Next, we used the publicly available metagenomics analysis software Taxonomer to screen the data sets for all known viral nucleotide and protein sequences [13]. This approach provided the opportunity to evaluate the usefulness of the NGS data and of the analysis tools for the determination of virus contamination. It also enables a thorough validation of the currently applied panel of PCR assays for the characterization and risk assessment of virus infections in cell lines.

## Materials and methods

### RNA-Seq and WES data

RNA-Seq and WES data of the analyzed cell lines (2 x 101 bp paired-end) were retrieved from Cancer Cell Line Encyclopedia (CCLE) via genetorrent from CGHub (https://cghub.ucsc.edu/software/downloads/GeneTorrent/3.8.7). In order to realign NGS data to virus sequences, the original downloaded bam files were sorted (samtools 0.1.19) and converted to fastq files (bedtools v2.21.0, BamToFastq). Reads were trimmed via fastq-mcf (ea-utils 1.1.2–686, fastq-mcf including homopolymer filtering and removing <Q20 flanking bases). Paired-end reads were joined where appropriate (ea-utils 1.1.2–686, fastq-join).

### Virus genomes

For the specific detection of virus sequences in the NGS data sets joined and unmerged sequence reads were aligned via STAR (2.5.2a, < = 10 mismatches, > = 20 bp chimeric alignments) to a combined genomic reference of the human genome (GATK hg38) and the virus genomes simultaneously. The virus sequences used were either the reference sequences or sequence data of representative virus strains (Table 1). All mapped reads were merged (samtools merged, 0.1.19) and counted for each contig given including human chromosomes and viral sequences (samtools idxstats).

**Table 1:** Viral genome reference used for detection of viral sequences in CCLE RNA-Seq and WES datasets.

Virus	Genome type*	Genome length	NCBI accession number; sequence type* or host cell line of isolate
Epstein Barr virus; human herpesvirus type 4 (EBV; HHV-4)	dsDNA	~172 kbp	NC_007605.1; NC_009334.1 (97% homol.); Ref.-Seq.
Hepatitis B virus (HBV)	dsDNA-RT	3,182 bp	NC_003977.2; Ref.-Seq.
Hepatitis C virus (HCV)	positive ssRNA	9,646 bp	NC_004102.1; Ref.-Seq.
Human herpesvirus type 8 (HHV-8)	dsDNA	~138 kbp	NC_009333.1; Ref.-Seq.
Human immunodeficiency virus type 1 (HIV-1)	ssRNA-RT	9,181 bp	NC_001802.1; Ref.-Seq.
Human immunodeficiency virus type 2 (HIV-2)	ssRNA-RT	10,358 bp	NC_001722.1; Ref.-Seq.
Human papillomavirus (HPV)	dsDNA	7,461 bp	NC_004500.1; Ref.-Seq.
Human T-lymphotropic virus type 1 (HTLV-1)	ssRNA-RT	8,507 bp	NC_001436.1; Ref.-Seq.
Human T-lymphotropic virus type 2 (HTLV-2)	ssRNA-RT	8,952 bp	NC_001488.1; Ref.-Seq.
Murine leukemia viruses (MLV)	ssRNA-RT	8,207 bp	AF221065.1; DG-75
Squirrel monkey retrovirus (SMRV)	ssRNA-RT	8,785 bp	NC_001514.1; Ref.-Seq.
Xenotropic murine leukemia virus related virus (XMRV)	ssRNA-RT	8,185 bp	FN692043.2; 22RV1

* dsDNA: double stranded DNA, dsDNA-RT: double stranded DNA with reverse transcribing RNA intermediate, ssRNA: single stranded RNA, ssRNA-RT: single stranded RNA with reverse transcription, Ref.-Seq: reference sequence, NCBI: National Center for Biotechnology Information, homol.: homology.

### Virus detection via Taxonomer

Trimmed and/or untrimmed sequence data files in fastq format were adjusted to approximately five gigabyte (maximal file size recommended for Taxonomer) using the “head”-command (head–n 80000000 [filename.fastq] > [filename1.fastq]). This non-random selection of reads might have introduced a sequencing bias due to favoring the first tiles of the sequencing plate leading to different base calling qualities compared to the rest of the plate which could result in lower qualities and diminished mapping rate. As the aim of this study was to detect virus incidence rather than to quantitatively measure virus abundance, we applied the simple subsampling via “head”. Regarding untrimmed sequence files, this corresponded to 2.0 x 10^7^ reads or 8.0 x 10^7^ lines. These files were analyzed with Taxonomer, a k-mer-based interactive metagenomics sequence analysis tool accessed through a web interface on the IOBIO framework (Center for Genetic Discovery at the University of Utah, Salt Lake City, USA). The software classifies each NGS read to one of the categories human, bacteria, viruses, phages, fungi, PhiX (used as a control for Illumina sequencing runs), ambiguous, and unknown sequences. This procedure is called “binning” and compares the read sequences to various sequence databases optimized for rapid k-mer queries. Unlike the other categories, previously unclassified sequences which might originate from virus sequences are first translated into amino acid sequences by a six-frame translation. The resulting amino acid sequences are then aligned to viral protein sequences of the UniProt public databases. This subroutine is called “Protonomer” as distinct from the Taxonomer application. This additional step has the advantage that genetically variable viruses can be detected even if the nucleotide sequences do not fit applying the exact k-mer matching. This method also offers the opportunity to even detect novel viruses.

The program can be run in two different modes. The first one is the full analysis mode screening all reads of a given data set with a file size limitation of five gigabyte corresponding to approximately 20 million reads of ca. 100 bp in length. The second mode is a quick analysis screening in which only a subset of 200,000 reads is analyzed. Selected reads identified by Taxonomer as virus specific were verified by a BLASTn comparison [14] to the National Center for Biotechnology Information (NCBI) nucleotide collection (nr/nt) database using MegaBLAST with the default parameters.

Five data sets of cell lines infected with viruses (as shown by PCR) and 25 data sets of cell lines not infected with viruses (according to PCR analysis) were randomly selected and trimmed to remove poor quality and adaptor sequences using Trimmomatic v0.36 [15] and FastQC v0.11.5 for quality evaluation. The Trimmomatic tool was run for Phred +33 files and we chose a sliding window of four nucleotides and a mean Phred score (Q value) of greater than 30 (SLIDINGWINDOW:4:30) corresponding to a mean P error of 0.001 over the four nucleotides. Furthermore, each sequence had to be longer than 80 nucleotides (MINLEN:80).

### PCR detection

Total nucleic acid was extracted from PBS-washed cell pellets of cell lines using the High Pure PCR Template Preparation kit (Roche, Mannheim, Germany) according to the recommendations of the manufacturer. The DNA of ca. 5 x 10^6^ viable cells was eluted in 200 µl dH_2_O. The DNA concentration and purity was determined using a Nanodrop 1000 fluorometer (Peqlab, Erlangen, Germany). Approximately 200 to 500 ng of the isolated DNA were used for the PCR amplification.

*Bovine polyomavirus (BPyV) detection of the SK-BR-3 cell line*. The forward primer was described previously [16] (QB-F1-1: 5´-CTAGATCCTACCCTCAAGGGAAT-3´) whereas the reverse primer BPyV-rev was newly designed according to the NCBI reference sequence (NC_001442.1): 5´-CTGACCTCCTCAACCTGTTTATC-3´. The PCR product is 422 bp long. One U TaKaRa Taq HS polymerase (Takara Bio Europe, Saint-Germain-en-Laye, France) and the respective 10x buffer (containing 15 mM MgCl_2_) were used in a 25 µl reaction mix containing 0.2 mM of each deoxynucleotide and 0.4 µM of each primer for the amplification. The hot start PCR was started with a Taq HS polymerase activating step at 94°C for 2 min. Subsequently, 35 cycles with denaturation steps at 94°C for 5 s, annealing steps at 57°C for 10 s, and amplification steps at 72°C for 20 s (plus 2 s extension for each cycle) were run. The amplified products were analyzed by agarose gel electrophoresis and visualized by ethidium bromide intercalation. The PCR product was then isolated from the reaction mix using a PCR product isolation kit (Qiagen, Hilden, Germany). For lack of a positive control for BPyV, the purified PCR product was sent away for sequencing (Eurofins Genomics, Munich, Germany). The DNA sequence was identified by a BLASTn alignment to the NCBI nucleotide (nr/nt) database (for parameters see paragraph on ´Virus detection via Taxonomer´).

*HTLV-1 detection*. A mixture of oligonucleotides was used for the PCR assay: forward SK110 (5´-CCC TAC AAT CCA ACC AGC TCM G-3´, 5´-CCT TAC AAT CCA ACC AGC TCA G-3´, and 5´-CCA TAC AAC CCC ACC AGC TCA G-3´); reverse SK111 (5´-GTG RTG GAT TTG CCA TCG GGT T-3´ and 5´-GTG GTG AAG CTG CCA TCG GGT T-3´). The PCR product is 182 bp long. The PCR was performed as described for the BPyV detection, but 30 cycles were run at 94°C denaturation temperature for 4 s, primer annealing at 60°C for 8 s, and amplification occurred at 72°C for 16 s plus 1 s extension for each cycle. The amplified products were identified by agarose gel electrophoresis and visualized by ethidium bromide intercalation. Genomic DNA of the HTLV-1 positive cell line MT-1 was used as a positive control.

## Results and discussion

### Direct comparison of PCR-based virus detection and RNA-Seq data-set analysis

In previous studies, we described the detection of virus infections in human and non-human primate cell lines by PCR assays [10, 17]. To demonstrate the applicability of NGS data for the detection of viruses in cell culture samples, we screened all cell lines previously shown by PCR to be virus infected and whose RNA-Seq (n = 20) or WES (n = 5) data are publicly available in the CCLE database [18]. The RNA-Seq and WES data were aligned against the respective complete viral genomes available in GenBank as listed in Table 1. Additionally, we chose 111 RNA-Seq as well as 58 WES data sets of predominantly leukemia and lymphoma cell lines which were PCR-negative for the panel of tested viruses to assess the accuracy of the alignment results (Tables 2 and S1).

**Table 2:** Number of RNA-Seq reads showing complete homology to viral reference sequences.

Cell Line	EBV	HBV	HCV	HHV-8	HIV-1	HIV-2	HPV	HTLV-1	HTLV-2	MLV	SMRV	XMRV	TotalReads
22RV1*	-	-	1	-	-	-	-	-	-	55660	-	1.2E+07	85721150
697	1	-	-	-	-	-	-	-	-	28	-	8	81259088
ALL-SIL	-	4	-	-	-	-	-	-	-	25	-	19	70640858
AML-193	1	-	-	-	-	-	-	-	-	44	-	18	46212784
BL-41	8	-	-	3	-	-	-	-	-	468	-	44	74420522
BL-70	22	-	-	13	-	-	-	-	-	459	-	44	77279657
BV-173	13	-	1	4	-	-	-	-	-	389	-	25	74710421
CA-46	19	-	-	11	-	-	-	-	-	768	-	94	78884064
CI-1*	140047	-	-	16	-	-	-	-	-	535	-	39	87224255
CMK	17	-	-	3	-	-	-	-	-	485	-	47	79388946
CML-T1	20	-	-	11	-	-	-	-	-	485	-	52	82236276
DAUDI*	173727	-	1	10	-	-	-	-	-	475	-	51	97286299
DB	-	-	-	-	-	-	-	-	-	11	-	622	76418339
DEL*	38	-	-	27	-	-	-	-	-	610648	-	110690	72128896
DOHH-2	724	-	-	17	-	-	-	-	-	440	-	35	87865712
DU-145	-	-	-	-	-	-	-	-	-	98	-	3447	33503822
EB-1*	575192	-	-	-	-	-	-	-	-	203	-	140	86441233
EHEB*	285258	-	-	3	-	-	-	-	-	552	-	50	78419270
EM-2	8	-	-	7	-	-	-	-	-	452	-	51	86296920
EOL-1	32	-	1	6	-	-	-	1	-	619	-	72	96434401
F-36P	-	-	-	-	-	-	-	-	-	35	-	13	73180415
GDM-1	30	-	-	3	-	-	-	-	-	606	-	45	70409443
GRANTA-519*	185588	-	2	11	-	-	-	-	-	427	-	30	66456317
HD-MY-Z	36	-	-	-	-	-	-	-	-	212	-	132	87315885
HEL	7	-	-	-	-	-	-	16	16	-	-	-	74625369
HEP-3B*	15	2721	1	-	-	1	-	9	-	1	-	-	71220537
HEP-G2	19	-	-	2	-	-	-	-	-	171	-	108	70787624
HH	5	-	1	-	-	-	-	27	-	-	-	-	82296966
HL-60	-	-	-	-	-	-	-	-	-	34	-	11	63088096
HPB-ALL	12	-	-	1	-	-	-	146	-	-	-	-	91740442
HT	4	-	1	1	-	-	-	40	-	-	-	-	85929580
JEKO-1	4	1	1	-	-	-	-	20	-	2	-	-	77907266
JK-1	6	-	-	-	-	-	-	-	-	63	50	35	54297156
JURKAT	9	-	-	-	-	-	-	-	-	103	169	48	60495191
JURL-MK1	5	-	-	-	-	-	-	-	-	128	87	91	59607922
JVM-2*	119506	-	-	1	-	-	-	-	-	75	71	37	53514535
JVM-3*	307211	-	-	-	-	-	-	-	-	64	83	40	64079997
K-562	1	-	-	-	-	-	-	-	-	-	-	-	69584223
KARPAS-299	27	-	-	-	-	-	-	-	-	78	88	41	55407660
KARPAS-422	6	-	-	1	-	-	1	-	-	51	65	30	59950611
KASUMI-1	-	1	-	-	-	-	-	-	-	20	-	36	70599398
KASUMI-2	15	-	-	-	-	-	-	-	-	91	91	57	52750315
KASUMI-6	12	-	-	-	-	-	-	-	-	114	95	64	65208346
KCL-22	-	-	-	-	-	-	-	-	-	88	96	57	60335903
KE-37	18	-	2	-	-	-	-	-	-	72	87	44	65753149
KELLY*	6	-	-	-	-	-	-	-	-	353930	90	201911	54463198
KG-1	8	-	-	-	-	-	-	-	-	93	64	53	61631553
KI-JK	7	-	-	1	-	-	-	-	-	850	64	753	65326438
KM-H2	-	-	-	-	-	-	-	-	-	-	-	-	26308903
KOPN-8	3	-	-	-	-	-	-	-	-	77	69	66	59680532
KU-812	13	-	-	-	-	-	-	-	-	80	70	66	61868133
KYO-1	3	-	-	-	-	-	-	-	-	93	92	49	54608638
KYSE-520	8	-	-	2	-	-	-	-	-	78	54	43	66550001
KYSE-70*	2	-	-	1	-	-	-	-	-	222376	108	175906	69815373
L-1236	7	-	1	-	-	-	-	-	-	140	51	83	68258990
L-428	11	-	-	2	-	-	-	-	-	74	90	53	66028849
L-540	9	-	-	1	-	-	-	-	-	73	67	44	53061075
LAMA-84	-	-	-	-	-	-	-	-	-	28	-	17	72590880
LOUCY	5	-	-	-	-	-	-	-	-	78	60	39	56263500
LXF-289*	17	-	-	1	-	-	-	-	-	133055	50	18334	66913976
M-07e	10	-	-	-	-	-	-	-	-	72	75	30	63871987
MC-116	10	-	-	-	-	-	-	-	-	100	65	57	60686609
ME-1	4	-	-	-	1	-	-	-	-	72	67	36	58761075
MEC-1*	64795	-	2	2	-	-	-	-	-	224	-	151	72193736
MEG-01	30	-	1	-	-	-	-	-	-	237	-	156	67020990
MHH-CALL-2	3	-	-	2	-	-	-	50	-	26	66	5	98588807
MHH-CALL-3	5	-	-	1	-	-	-	59	-	17	49	11	85108243
MHH-CALL-4	-	-	-	-	-	-	-	166	-	15	40	4	65284221
ML-1*	1	-	-	-	-	-	-	49	1	674129	52	169661	94044711
MOLM-13	13	-	1	-	-	-	-	-	-	133	-	94	58974117
MOLM-16	7	-	-	-	-	-	-	45	-	19	66	-	93280048
MOLM-6	5	-	-	-	11	-	-	51	-	13	28	4	90804125
MOLT-13	4	-	1	-	-	-	-	40	-	23	80	5	77681738
MOLT-16	12	-	1	-	-	-	-	60	-	22	79	2	82739072
MOLT-3	3	-	3	-	-	-	-	63	-	22	68	6	96080882
MONO-MAC-1	-	-	-	-	-	-	-	-	-	38	-	10	63870267
MONO-MAC-6	-	-	-	1	-	-	-	-	-	24	-	28	80678498
MSTO-211H	-	-	-	-	-	-	-	-	-	-	-	-	55249182
MUTZ-3	2	-	-	-	-	-	-	31	-	10	31	4	42366029
MUTZ-5	11	-	-	-	-	-	-	45	-	23	172	9	90207271
MV4-11	24	-	-	-	-	-	-	-	-	175	-	114	82669111
NALM-19	13	-	-	1	-	-	-	62	-	29	92	4	99690603
NALM-1	22	-	-	2	-	-	-	60	-	11	68	8	87993249
NALM-6	1	-	-	-	-	-	-	-	-	35	-	8	66567771
NAMALWA*	33388	-	1	-	-	-	-	52	-	27	2.9E+06	4	96154122
NB-4	-	-	-	-	-	-	-	-	-	52	2	12	75735056
NOMO-1	12	-	-	-	-	-	-	-	-	22	-	7	89497585
NU-DHL-1	2	-	-	-	-	-	-	86	-	15	81	-	89167696
NU-DUL-1	1	-	-	-	-	-	-	36	-	10	75	6	73436859
OCI-AML2	-	-	-	-	-	-	-	-	-	48	-	13	75916480
OCI-AML3	-	-	-	-	-	-	-	-	-	24	-	8	67132643
OCI-AML5	-	3	-	1	-	-	-	-	-	22	-	5	75076488
OCI-LY19	6	-	-	-	-	-	-	43	-	25	62	5	84358650
OCI-M1	5	-	-	-	-	-	-	-	-	207	-	121	64914233
P12-ICHIKAWA	8	-	-	-	-	-	-	75	-	39	72	7	101732384
PEER	-	1	1	-	-	-	-	-	-	5	-	4	86530574
PF-382	5	-	-	-	-	-	-	-	-	9	-	2	79902288
PL-21	6	-	-	-	-	-	-	-	-	6	-	-	90057568
RAJI*	84154	1	-	-	-	-	-	-	-	8	-	7	93092565
REC-1	11	-	-	-	-	-	-	-	-	8	-	8	92336422
Reh	-	2	-	-	-	-	-	-	-	28	-	6	64967602
RI-1	3	3	-	-	-	-	-	-	-	11	-	1	90836361
RL	23	-	-	-	-	-	-	-	-	163	-	87	65102089
RPMI-8402	3	-	1	-	-	-	-	-	-	8	-	4	80955217
RS4-11	-	1	1	-	-	-	-	-	-	34	-	15	63580953
S-117	3	-	-	1	-	-	-	-	-	196535	-	106742	82999294
SCLC-21H*	1	-	-	1	-	-	-	-	-	505605	1	343039	75852742
SEM	-	2	-	-	-	-	-	-	-	29	-	10	65698069
SET-2	16	-	-	-	-	-	-	-	-	165	-	127	69737227
SIG-M5	11	2	1	1	-	-	-	-	-	1	-	1	73177347
SIMA	2	4	-	-	-	-	-	-	-	2	-	2	84105555
SKM-1	5	-	-	-	-	-	-	-	-	5	-	4	90202981
SK-MEL-1*	6	-	-	-	-	-	-	-	-	3	-	6	87914334
SK-MEL-30	1	-	-	-	-	-	-	-	-	26	-	6	67161539
SK-MES-1	-	2	-	-	-	-	-	-	-	23	-	7	69037222
SR-786	-	-	-	-	-	-	-	-	-	51	-	57	97172481
SU-DHL-10	-	-	1	-	-	-	-	-	-	17	-	15	71195074
SU-DHL-1	-	-	-	-	-	1	-	-	-	22	-	37	80321452
SU-DHL-4	-	2	-	-	-	-	-	-	-	25	-	30	75321327
SU-DHL-5	-	-	-	1	-	-	-	-	-	53	-	74	34529760
SU-DHL-6	-	-	-	-	-	-	-	-	-	83	-	104	67909179
SU-DHL-8	-	-	-	-	-	-	-	-	-	23	-	14	86339964
SUP-B15	2	-	-	-	-	-	-	-	-	27	-	37	79116194
SUP-M2	9	-	2	1	-	-	-	-	-	234	-	161	70455526
SUP-T11	11	-	-	-	-	-	-	-	-	54	-	55	78844477
SUP-T1	-	-	-	-	-	-	-	-	-	24	-	31	67595481
TALL-1	-	2	1	-	-	-	-	-	-	30	-	16	53297679
TF-1	-	-	-	-	-	-	-	-	-	15	-	10	75865774
THP-1	-	1	-	-	-	-	-	-	-	37	-	10	69965630
U-937	16	-	-	1	-	-	-	-	-	152	-	109	72515678
WSU-DLCL2	-	3	-	-	-	-	-	-	-	31	-	35	32947165

* Cell lines previously shown by PCR to be virus infected. For information on the cell lines and the infections determined by PCR, please refer to S2 Table.

As shown in Table 2, the sequencing depth of the RNA-Seq data sets varied between 2.6 x 10^7^ reads and 1.0 x 10^8^ reads with an average of 7.3 x 10^7^ reads. This overall number of sequenced reads usually allows the quantification of transcripts with low levels of expression [19]. As the aim of our RNA-Seq analysis is to qualitatively detect any viral gene expression and not the exact determination of differential expression levels, no further normalization procedures were done. On the other hand, a very high sequencing depth might lead to the formation of transcriptional noise. Besides the sequencing depth the type of RNA preparation (e.g. total RNA, mRNA, including small RNAs) and transcript length are important for the representation of sequences within the RNA-Seq data sets. Most of the protein coding mRNAs are well represented in the reads of moderate sequencing depth (5 x 10^7^ reads) whereas small RNA species are less represented in those data sets, but their relative abundance increases with ultra-high-throughput sequencing (1 x 10^8^ reads) [20]. Viruses affect the physiology of infected cells, regardless of the infection status. Thus, the viral genome is at least in part transcribed to produce regulatory active RNAs (e.g. miRNAs, lncRNAs) or mRNAs for protein expression. All these RNAs are polyadenylated, isolated with poly-A+ RNA enrichment and should be well represented in the data sets. Moreover, as we already had obtained data regarding the virus infection status of the investigated cell lines by PCR, we were able to use those results as valid reference to evaluate the results of the NGS data analyses.

Regarding the cell lines positive for virus infections as shown by PCR all virus specific reads ranged from approximately 2,700 reads (HBV of HEP-3B), corresponding to 0.00382% of all reads and up to 1.2 x 10^7^ reads (XMRV of 22RV1) corresponding to as many as 14% of all reads. The median number of virus specific reads is 196,535 at a median total reads number of 7.8 x 10^7^. This corresponds to 0.25% of the total reads number. No virus specific reads were detected for the vast majority of the cell lines for HBV, HCV, HIV-1 and -2, and HPV infections. Overall, 17 cell lines showed one to four HBV specific reads, 25 cell lines up to three HCV reads, two cell lines one and 11 HIV-1 reads, two cell lines with one HIV-2 read each, one cell line with one HPV read, and two cell lines with one and 16 HTLV-2 reads. HHV-8 specific reads of 39 cell lines were predominantly also very low, but with single samples showing up to 27 reads.

Regarding HTLV-1, 25 cell lines showed a notable number of up to 166 (average: 53) aligned reads. Although the PCR positive cell lines showed a minimum read number that was more than fifteenfold, we have further analyzed the individual read sequences for HTLV-1 specificity. The qualities of the reads are good or even excellent according to the Phred-Score values of each nucleotide of the sequences. A subsequent BLASTn analysis of the reads revealed 98–100% nucleotide identity for HTLV-1 sequences listed in the public databases. Furthermore, the sequences represent regions of the complete proviral HTLV-1 genome. Table 3 exemplifies alignments of a few HTLV-1 specific reads of the MHH-CALL-4 cell line. Thus, we conclude that the reads are indeed present in the samples and that they were correctly analyzed and assigned.

**Table 3:** HTLV-1 reads aligned to HTLV-1 sequences of the NCBI sequence data base.

Read name / NCBI accession number	Sequence alignment	Nucleotide numbers
C1DVPACXX130111:8:1206:15932:77870/ MG493464.1	GACGTGTCCCCCTGAAGACAAATCATAAGCTCAGACCTCCGGGAAGCCACCGGAACCACCCATTTCCTCCCCATGTTTGTCAAGCCGCCCTCAGGCGTTGACGACAACCCCT	1–112
	................................................................................................................	159–270
	CACCTCAAAAAACTTTT	113–129
	.................	271–287
C1DVPACXX130111:8:2102:7640:89060/ MF277085.1	TCTCACACGGCCTCATACAGTACTCTTCCTTTCATAATTTACATCTCCTGTTTGAAGAATACACCAACATCCCCATTTCTCTACTTTTTAACGAAAAAGAGGCAGATGACAA	1–112
	...............................................................................................................	8174–8285
	TGACCATGAGC	113–123
	...........	8286–8296
C1EHHACXX130117:6:1101:7974:95240/ LC192519.1	CTCCCAGTGAAAAACATTTCCGCGAAACAGAAGTCTGAAAAGGGCAGGGCCCAGACCAAGGCTCTGACGTCTCCCCCCGGAGGGACAGCTCAGCACCGGCTCAGGCTAGGCC	1–112
	...........................................T....................................................................	8324–8435
	CTGACGTGTCCCCCTGAAGACAAATCATAAGCTCAGACCTCCGGGAAGCCACCGGAACCACCCATTTCCT	113–182
	......................................................................	8436–8505
C1FBVACXX130129:3:1102:17215:64814/ KC807984.1	CTCCGTTGTCTGCATGTACCTCTACCAGCTTTCCCCCCCCATCACCTGGCCCCTCCTGCCCCACGTGATTTTTTGCCACCCCGGCCAGCTCGGGGCCTTCCTCACCAATGTT	1–112
	................................................................................................................	7745–7856
	CCCTACAAGCGAATAGAAGAACTCCTCTATAAAATTTCCCTTACCACAGGGGCCCTG	113–169
	.........................................................	7857–7913
C1EHHACXX130117:5:2310:16190:61202/	GCTGTTTCGCCTTCTCAGCCCCTTGTCTCCACTTGCGCTCACGGCGCTCCTGCTCTTCCTGCTTTCTCCGGGCGACGTCAGCGGCCTTCTTCTCCGCCCGCCTCCTGCGCCG	1–112
LC378575.1	................................................................................................................	6838–6949
C1FBVACXX130129:3:1103:2255:58304/	ATCGAGTCTTGACTGGCTGGGGCCTTAACTGGGACCTTGGCCTCTCACAGTGGGCTCGAGAGGCCTTACAAACTGGAATCACCCTTGTCGCGCTACTCCTT	1–101
LC210065.1	.....................................................................................................	6447–6557
C1EHHACXX130117:1:2114:20232:93144/	GCGGCCGTCATGGCCCTTTCAGCAGATCAGGCCCGACAGCCCCCCGGCCCTAATCTAGTAGGTTACTCTAGCTACCATGCCACCTATTCCCTATATCTATTCCCTCG	1–107
LC183872.1	..................................T..........T.............................................................	5345–5440
C1EHHACXX130117:8:1311:16345:79129/ LC192533.1	CGAGAATACCAGCAACTCTGGCTCGCCGCCTTCGCCGCCCTGCCAGGGAGTGCCAAAGACCCTTCCTGGGCCTCTATCCTCCAAGGCCTGGAGGAGCCTTACCACGCCTTCG	1–112
	................................................................................................................	1524–1635
	TAGAACGCCTCAAC	113–126 1636–1649
	..............	

We then tried to validate the results with a verification of the initial PCR analyses. If the cell line is infected with HTLV-1, each of the cells in the culture should contain at least one copy of the virus as provirus integrated into the genome. If only a few cells express RNA as detected by RNA-Seq analysis, PCR of the genomic DNA of the cell line should be able to amplify the HTLV-1 sequence. However, no HTLV-1 specific sequences were amplified confirming the initial test results. Moreover, we performed RNA-Seq of our own HPB-ALL cells (DSMZ ACC 483) which are listed with 146 HTLV-1 reads in the CCLE data-set. Analyzing the reads with the same method as the CCLE RNA-Seq data revealed no HTLV-1 specific sequences.

While arranging the cell lines according to their accession numbers, we found that 24 apparently HTLV-1 containing cell lines clustered in one single group from G28530.MUTZ-5 to G28888.Hep_3B2.1–7 (Table 4). Only G27380.EOL-1 with a single HTLV-1 specific read was located outside this cluster. Due to their origin and cell type, it seems to be very unlikely that most of the HTLV-positive cell lines are in fact infected with HTLV-1 ab initio. Thus, we conclude that the retrovirus-specific mRNAs were caused by a contamination during RNA extraction or library preparation, assuming that the cell lines were analyzed in the same laboratory and that the RNAs were prepared with the same extraction method. This implication is supported by the observation that the magnitude orders of other viruses also show a significant clustering among the other low level virus-specific reads. This is obvious for 15 cell lines regarding HHV-8 which arrange similarly as HTLV-1, but with 3 to 27 HHV-8 specific reads per cell line, forming a consistent set of cell lines with low level reads from G27318.DEL to G27390.CMK. A third clear clustering concerns SMRV detection. Forty-four cell lines display contamination caused reads in the range of 28 to 172 specific reads interrupted by one cell line with no specific reads (KM-H2) and one SMRV positive cell line (NAMALWA) (Table 4). Although almost all cell lines display virus-specific reads for MLV, interestingly, similar magnitudes of reads cluster in separate sets (data not shown).

**Table 4:** HTLV-1, HHV-8, and SMRV specific read numbers of cell lines ordered by CCLE file names.

HTLV-1	File name*	HHV-8	File name*	SMRV	File name*
-	G28080.JURKAT.1.bam	1	G27258.OCI-AML5.1.bam	50	G28000.JK-1.1.bam
-	G28082.KELLY.1.bam	-	G27288.ALL-SIL.1.bam	91	G28002.KASUMI-2.1.bam
-	G28086.KOPN-8.1.bam	-	G27290.RS4_11.1.bam	60	G28006.Loucy.1.bam
45	G28530.MUTZ-5.1.bam	27	G27318.DEL.1.bam	75	G28007.M-07e.1.bam
62	G28531.NALM-19.1.bam	7	G27324.EM-2.1.bam	71	G28008.JVM-2.1.bam
166	G28532.MHH-CALL-4.1.bam	13	G27333.BL-70.1.bam	87	G28009.KE-37.1.bam
36	G28540.NU-DUL-1.1.bam	11	G27335.GRANTA-519.1.bam	87	G28010.JURL-MK1.1.bam
49	G28541.ML-1.1.bam	11	G27344.CML-T1.1.bam	65	G28013.KARPAS-422.1.bam
86	G28542.NU-DHL-1.1.bam	4	G27347.BV-173.1.bam	90	G28022.L-428.1.bam
43	G28543.OCI-LY-19.1.bam	10	G27352.Daudi.1.bam	64	G28028.KG-1.1.bam
51	G28544.MOLM-6.1.bam	11	G27359.CA46.1.bam	51	G28032.L-1236.1.bam
50	G28551.MHH-CALL-2.1.bam	16	G27371.CI-1.1.bam	92	G28037.KYO-1.1.bam
45	G28555.MOLM-16.1.bam	3	G27374.BL-41.1.bam	-	G28039.KM-H2.1.bam
60	G28565.MOLT-16.1.bam	17	G27375.DOHH-2.1.bam	67	G28040.L-540.1.bam
60	G28567.NALM-1.1.bam	3	G27378.EHEB.1.bam	95	G28043.Kasumi-6.1.bam
40	G28576.MOLT-13.1.bam	6	G27380.EOL-1.1.bam	64	G28046.Ki-JK.1.bam
52	G28578.NAMALWA.1.bam	3	G27388.GDM-1.1.bam	50	G28047.LXF-289.1.bam
75	G28600.P12-ICHIKAWA.1.bam	3	G27390.CMK.1.bam	67	G28048.ME-1.1.bam
59	G28609.MHH-CALL-3.1.bam	1	G27458.SIG-M5.2.bam	96	G28049.KCL-22.1.bam
31	G28621.MUTZ-3.1.bam	-	G27463.SK-MEL-1.2.bam	54	G28054.KYSE-520.1.bam
63	G28623.MOLT-3.1.bam	-	G27474.RPMI-8402.2.bam	70	G28055.KU812.1.bam
146	G28828.HPB-ALL.3.bam	-	G27482.SIMA.2.bam	65	G28058.MC116.1.bam
40	G28835.HT.3.bam			88	G28063.KARPAS-299.1.bam
20	G28842.JeKo-1.3.bam			108	G28067.KYSE-70.1.bam
16	G28844.HEL.3.bam			83	G28068.JVM-3.1.bam
27	G28867.HH.3.bam			169	G28080.JURKAT.1.bam
9	G28888.Hep_3B2.1–7.3.bam			90	G28082.KELLY.1.bam
-	G30554.KASUMI-1.1.bam			69	G28086.KOPN-8.1.bam
-	G30556.SU-DHL-8.1.bam			172	G28530.MUTZ-5.1.bam
-	G30561.SUP-T1.1.bam			68	G28531.NALM-19.1.bam
				40	G28532.MHH-CALL-4.1.bam
				75	G28540.NU-DUL-1.1.bam
				52	G28541.ML-1.1.bam
				81	G28542.NU-DHL-1.1.bam
				62	G28543.OCI-LY-19.1.bam
				28	G28544.MOLM-6.1.bam
				66	G28551.MHH-CALL-2.1.bam
				66	G28555.MOLM-16.1.bam
				79	G28565.MOLT-16.1.bam
				92	G28567.NALM-1.1.bam
				80	G28576.MOLT-13.1.bam
				293571	G28578.NAMALWA.1.bam
				72	G28600.P12-ICHIKAWA.1.bam
				49	G28609.MHH-CALL-3.1.bam
				31	G28621.MUTZ-3.1.bam
				68	G28623.MOLT-3.1.bam

*according to https://portal.gdc.cancer.gov/legacy-archive/search/f selected for “Cancer Program: CCLE”

EBV is the only virus of which the low number reads are almost constantly distributed over all cell lines. Ninety-six cell lines revealed EBV specific reads of which 85 cell lines showed read numbers of one to a maximum of 38 reads in the cell line DEL. The other cell lines were EBV positive according to PCR analysis with read numbers between 33,388 (NAMALWA) and 575,192 (EB-1) demonstrating the enormous difference between true positive samples and samples with viral contamination caused reads.

Only the cell line DOHH-2 revealed a notably higher, although compared to PCR positive samples still very low number of EBV specific reads (n = 724). We have previously shown by fluorescence in situ hybridization that the DOHH-2 cell line consisted of two clones: most of the cells were clearly EBV negative, whereas a few cells contained several EBV episomes within the nucleus [17]. Single cell cloning was performed to separate the EBV-negative from the EBV-positive cells. The original publication describing the establishment of the DOHH-2 stated that the cell line was negative for the Epstein-Barr virus nuclear antigen type 1 (EBNA-1) [21]. The relatively low number of EBV-specific reads compared to all other EBV positive cell lines can be attributed to the original mixed culture containing a low portion of EBV positive cells. This result is in concordance with the findings of Cao et al. [22] who also determined low level expression, but significantly above the contamination caused level.

Regarding the animal retroviruses MLV, XMRV, and SMRV, a large number of cell lines revealed virus specific sequence reads. In general, MLV and XMRV showed a higher magnitude of the contamination caused reads per cell line (up to 850 for KI-JK) compared to the low level reads of other investigated viruses. Moreover, specific reads were found in a high proportion of the cell lines (116 low level positive cell lines of 124 cell lines with XMLV reads and 111 low level of 119 cell lines with XMRV reads). The similar number of cell lines with MLV and XMRV specific reads is attributed to the high relatedness of the genomes because XMRV is in fact a hybrid of two different XMLV strains [23]. Regarding SMRV, 47 cell lines exhibit of up to 172 specific reads for that virus. In contrast to those numbers the clearly SMRV positive NAMALWA cell line exhibits almost 3 x 10^6^ specific reads. Although not as striking as in the case of HTLV-1, all these reportedly positive cell lines cluster in sets with higher or lower numbers of virus specific reads. In our opinion, all these examples support the explanation that the low count virus specific reads are artifacts which can be attributed to contaminations in the course of RNA extraction or during further processing steps. For instance, multiple cell line sequencings performed on the same sequencing run (sample multiplexing) may lead to index misassignment (cross-talk) due to inappropriately matched indices of the library pool to sequence reads which were derived from different samples in the pool. This can be virtually eliminated using dual-matched indexed adapters containing an additional “Unique Molecular Index” (UMI) appended to the 3´ end of the i7 index for molecule barcoding [24].

Several studies have investigated the assignment of unmapped reads in NGS data. According to Tae et al. 0.13% of the reads in a subset of 150 genomes of the 1000 Genomes Project showed similarities to non-human genomes [25]. Although they investigated WGS and WES, they found that different sequencing centers had specific signatures of contaminating genomes as ´time stamps´. Strong et al. describe a similar finding regarding RNA-Seq data sets from The Cancer Genome Atlas (TCGA) [26]. They analyzed 244 different specimens from different sources and from different specimen types and found average numbers ranging from 1406 reads per million human mapped reads (RPMHs) to 11,106 RPMHs with taxa-specific differences across centers. Furthermore, identical cell lines analyzed in separate studies showed differences in bacterial read profiles. R.W. Lusk claims that “contamination must be considered a potential source of signals of exogenous species in sequencing data, even if these signals are replicated in independent experiments, vary across conditions, or indicate a species which seems a priori unlikely to contaminate” [27]. Another indication that contaminations might be prevalent in nucleotide samples for NGS projects was shown in our previous study detecting XMLV contamination in human cell lines. Four out of 23 PCR-positive cell lines turned out to be false positive due to previously unrecognized DNA contamination with mouse DNA introducing the endogenous XMLV sequences into the sample. The contamination was verified by the amplification of intracisternal A particles (IAP) which are present at ca. 1,000 copies per haploid mouse genome. The low level contaminations occurred when mouse DNA was extracted simultaneously with or previously to the human DNA [10]. Contamination of NGS samples with mouse DNA or RNA might be an explanation for the widespread occurrence of the retroviral sequences in the NGS data sets.

Taken together, virus infections of cell lines can easily and with high specificity and sensitivity be detected in RNA-Seq data sets. However, low numbers of virus specific reads are likely to be the result of contaminating nucleic acids rather than an indication of viral infections in cell lines. The identification of contaminating RNA or DNA appears to be difficult because minimal amounts of the nucleic acids result in the generation of the few reads and they can only be detected if they are not a genuine part of the human transcriptome and can be mapped as in the current study. If the contamination is of human origin, identification appears to be impossible. Performing RNA preparations and sequencing reactions in parallel with control samples known to be free of contaminations and subsequent alignment against an artificial chromosome of the different virus sequences may give evidence of a contamination. A strict separation of RNA preparation for NGS and other molecular biology experiments seems to be mandatory. In the current study we were able to identify contaminated cell lines by means of verification with an independent PCR assay and the arrangement of numerous data sets. Defining thresholds for individual viruses appears to be difficult but for cell lines read numbers of up to 1,000 might be attributed to contaminations. But this could be different for primary tumor samples or other cell samples. However, the HBV infected cell line HEP-3B exhibited less viral reads (n = 2,695) than the DU-145 cell line which is PCR negative for MLV but shows 3,447 MLV reads. The RNA extraction and library preparation of cell lines with such high numbers of virus specific reads should be repeated, if possible, or a possible infection should be verified with another method.

### Direct comparison of PCR-based virus detection and WES data set analysis

WES data are available for a number of cell lines in the CCLE database. We have analyzed the sequences of 62 data sets of continuous cell lines in an identical manner as the RNA-Seq data sets. The total read numbers of the samples were in the same range as the RNA-Seq data: from approximately 3.0 x 10^7^ reads to about 1.02 x 10^7^ reads, representing a high coverage of the exome. As WES reads are generated with exon specific primers, virus specific sequences are generally off-target sequences and part of the sequences unmapped to the human genome. Therefore, for WES data sets virus specific sequences are generally expected at a lower proportion of virus specific reads relative to the total reads compared to a respective RNA-Seq data set. As shown in S1 Table, we analyzed the data sets of 62 cell lines matching RNA-Seq data sets. Of those, six cell lines were previously shown by PCR analysis to be virus infected. Of those virus infected cell lines only the HBV infection of the HEP-3B cell line was not detected. The HBV genome of the HEP-3B cell line is only about 2 kb in size. This sequence might be too short for a detection as an untargeted sequence even at high coverage. This might also be true for other viruses with small genomes such as parvoviruses, circoviruses and segmented viruses where individual segments may be only a few kilobases in length.

The number of virus specific reads detected in the data sets of the infected cell lines is substantially lower in WES compared to RNA-Seq results. RNA-Seq reveals ca. 30 to 91 times more EBV reads (4,583 versus 140,047 reads for CI-1 and 365 versus 33,388 reads for NAMALWA, normalized to the total number of reads) and even about 93,000 times more XMRV reads (129 versus 1.2 x 10^7^ reads for 22RV-1, normalized to the total number of reads). The least number of 34 virus specific reads was obtained for the cell line NAMALWA for SMRV (versus 3 x 10^6^ regarding RNA-Seq) and the cell line CI-1 exhibited the highest number of 2,144 virus specific reads for EBV. Except for HEP-3B, all cell lines previously found to be virus infected were also positive (4 x EBV, 1 x XMRV, 1 x SMRV) by sequence analysis. This clearly demonstrates that RNA-Seq data are more powerful regarding sensitivity, clarity and reliability for the detection of viruses in cell cultures than WES data.

On the other hand, the results of WES data were more specific. Except for 11 cell lines matching EBV unspecifically with 16 or less reads, only one unspecific HCV alignment was detected by this method (OCI-LY19). No other unspecific reads were listed.

Taken together, WES data sets are less qualified for virus detection in cell lines than RNA-Seq data sets. The detection accuracy highly depends on the quality of the sequencing procedure: highly specific annealing conditions for the sequencing primers decreases the number of off-target reads that do not map to the human genome and thus decreases the number of virus specific reads. Regarding publicly available data sets from different sequencing centers the quality standards might be different and the results might not always be directly comparable.

### Taxonomer

To address the general infection status of cell lines regarding viruses, we applied the publicly accessible analysis tool Taxonomer of IDbyDNA.

As mentioned before, we have previously analyzed all human and non-human primate cell lines for specific viral pathogens by PCR. Other viruses found to be present in cell cultures (e.g. MLV, XMRV, SMRV) were investigated for the characterization of the cell lines. However, apart from those viruses, we have never been certain whether or not other viruses were present in the cell lines originating from the patient or the original host or whether they might have been introduced later during cell cultivation (e.g. by FBS or other cell culture supplements or from specific treatments of the cells) [28].

As shown in the section on the evaluation of the RNA-Seq data for specific viruses in comparison with the findings using PCR detection, all contaminating viruses could be detected applying the NGS data sets. We chose 301 CCLE RNA-Seq data sets for a screening with the Taxonomer tool applying the quick analysis mode (S2 Table). All of the cell lines are concurrently part of the Leibniz Institute DSMZ cell lines bank and had been tested by PCR for virus infections.

In one approach a selection of 30 out of the 301 RNA-Seq data sets were trimmed to eliminate the low-quality sequences applying the Trimmomatic tool developed by Bolger et al. [15]. We chose a sliding window of 4 nucleotides and a mean Phred score (Q value) of greater than 30 (SLIDINGWINDOW:4:30) corresponding to a mean P error of 0.001 over the four nucleotides. Furthermore, each sequence had to be longer than 80 nucleotides (MINLEN:80).

**Fig 1:**
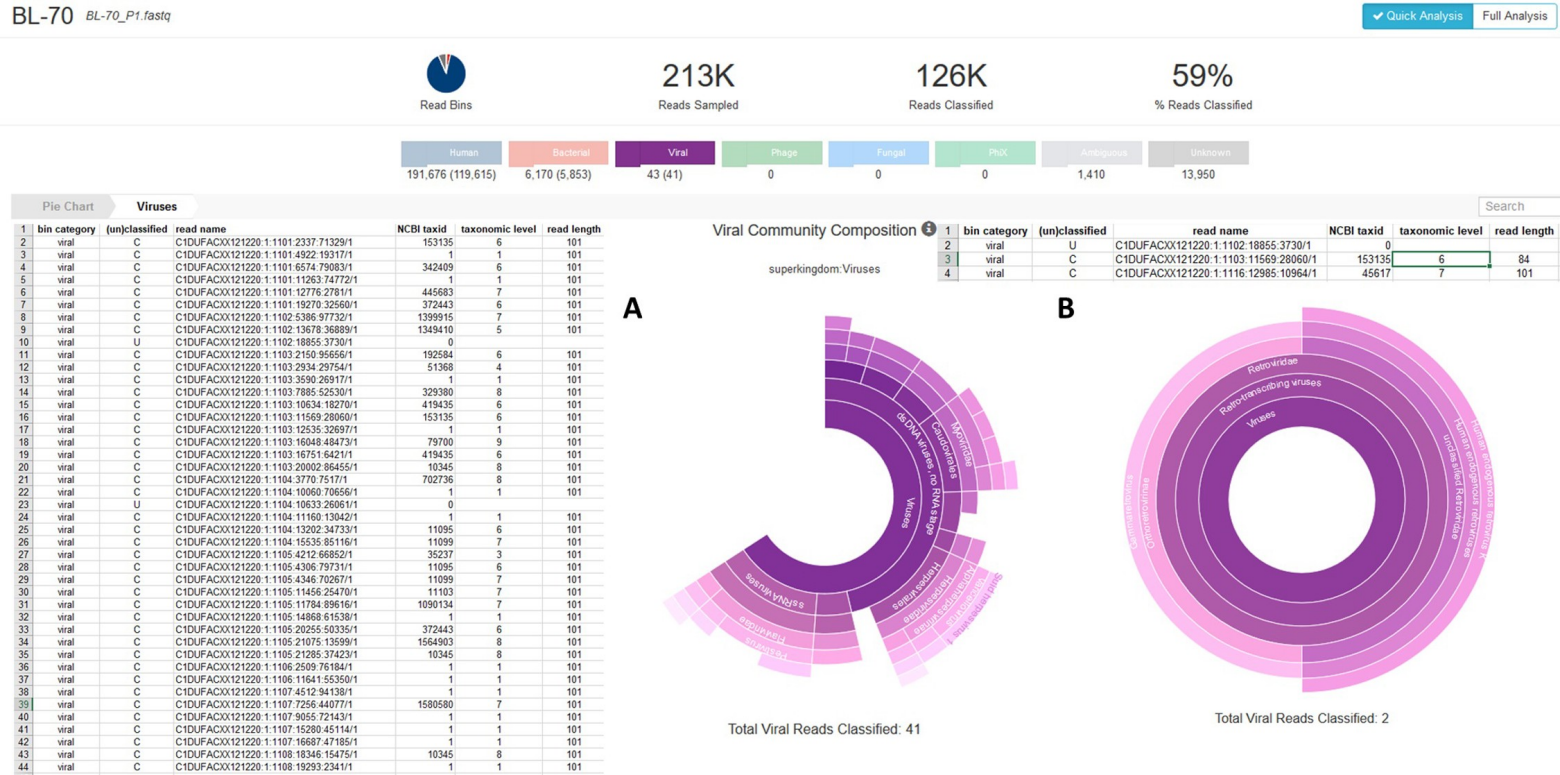
Virus detection in the cell line BL-70 applying the Taxonomer bioinformatics tool before and after trimming. Shown are analysis outputs of quick analyses of 213,249 reads. Of those, 125,509 reads (59%) were classified to the human, bacterial, and viral bins (numbers in barackets). A) The table lists all reads categorized to the viral bin before trimming. The reads with a taxonomic level higher than 1 are depicted in the circular diagram. B) The table lists the remaining viral reads after trimming the reads using the Trimmomatic tool (SLIDINGWINDOW:4:30; MINLEN:80) and the two classified viral reads in the circle.

Taking the BL-70 cell line as an example, applying this process to the first 20 million sequences of the RNA-Seq data set, the number of reads complying with the mentioned criteria was reduced to 6,076,955 reads (30.38%). The following Taxonomer analysis revealed three virus specific reads after binning and two reads after the additional Protonomer analysis (Fig 1B). The unclassified read was identified as XMLV. Of the two reads classified by Protonomer one was also identified as XMLV and the second classified read aligned to a human endogenous retrovirus K (HERV-K). Taking the number of XMLV reads determined by the virus genome specific analysis as a basis (447 out of 7.73 x 10^7^ reads, as mentioned in Table 2) and assuming an equal distribution of the XMLV sequences in the data set, two XMLV specific reads is exactly the number to be expected in the quick analysis. In fact, the sequences assigned to XMLV were two of the reads determined by the alignment of the XMLV genome sequences. Thus, the XMLV hits were correctly assigned by Taxonomer, although, as discussed before, we conclude that the detected reads originate from a contamination of the RNA sample.

Of the 30 cell lines selected for read trimming, four were positive for virus infections as confirmed by the other detection methods before. Table 5 shows that number of reads with viral sequences were almost constant before and after trimming regarding those virus positive cell lines (22RV1, DAUDI, DEL, MEC-1), indicating that most of the confirmed virus positive reads were specific and of good quality. On the other hand, regarding 14 cell lines with viral reads in the range of 10 to 50 before trimming none of the viral reads survived the trimming procedure and no further previously unconsidered reads were appended. The remaining cell lines perform similarly as the beforehand as example mentioned BL-70 lying in the range of one to five virus reads after trimming. Only cell line DU-145 revealed nine viral reads, all identified as retroviruses and eight of them were further classified as gammaretroviruses. The direct screening of the DU-145 reads for XMLV and XMRV complete genomes exhibits a relatively high number of XMRV reads (n = 3,447; see Table 2). Given a total read number of 3.35 x 10^7^, this would result in approximately one XMRV read per 10,000 reads. Hence, even about 20 reads might have been expected in the Taxonomer quick analysis of 2 x 10^5^ reads. The range of XMLV (XMRV) positive cell lines show read numbers of 261 to almost 13,000 classified reads, which is at least 30 times more than the contamination caused counts. Most of the viral reads after trimming were classified as XMLV (XMRV), but also single reads belonged to Feline sarcoma virus, Paramyxovirinae, and Pandoravirus salinus. None of the latter viruses was ever described to affect human continuous cell cultures. As discussed before, it is likely that these hits result from amplifications inherent to the NGS method or might be a result of homologous sequences between those viruses and the murine retroviruses.

**Table 5:** Comparison of read numbers assigned to the viruses and bacteria bins before and after trimming the RNA seq data sets.

Cell line	Before trimming			After trimming			Virus species identified by Taxonomer	Infection identified by PCR
	Overall virus reads	Reads after Protonomer	Reads of infecting virus species (according to PCR)	Overall virus reads	Reads after Protonomer	Reads of infecting virus species (according to PCR)		
22RV-1	14113	12495	11351	13418	11935	10882	XMLV / XMRV	XMRV
697	19	19	-	-	-	-		
AML-193	14	13	-	-	-	-		
BL-41	42	42	-	2	2	-	FSV / XMLV	
BL-70	43	41	-	3	2	-	HERV-K / XMLV	
CAL-27	11	9	-	5	4	-	Paramyxov. / XMLV	
DAUDI	280	203	146	190	127	127	EBV	EBV
DBTRG-05MG	14	14	-	-	-	-		
DEL	1184	982	858	1066	921	839	XMLV	XMLV
DK-MG	24	24	-	-	-	-		
DU-145	23	21	-	9	9	-	XMLV	
EFM-192A	41	40	-	-	-	-		
GAMG	33	33	-	1	1	-	XMLV	
GDM-1	42	42	-	1	1	-	XMLV	
HCC-827	34	34	-	-	-	-		
HCT-15	6	6	-	-	-	-		
HEP-3B	31	31	12	9	9	9	HBV	HBV
IGR-37	5	5	-	2	2	-	XMLV	
K-562	20	20	-	-	-	-		
KASUMI-1	15	15	-	-	-	-		
KMS-12BM	20	20	-	1	1	-	Pandoravirus	
KYSE-150	14	13	-	2	2		XMLV	
KYSE-180	5	5	-	-	-	-		
KYSE-270	9	9	-	1	1		XMLV	
LAMA-84	21	21	-	-	-	-		
MEC-1	189	151	133	160	123	116		EBV
MFE-296	7	7	-	-	-	-		
MHH-CALL-4	13	13	-	2	2	-	Mastadenov. / DTP	
NB-4	30	30	-	-	-	-		
NOMO-1	24	24	-	-	-	-		

Abbreviations: DTP: Deep-sea thermophilic phage D6E, FSV: Feline sarcoma virus, HERV-K: Human endogenous retrovirus type K, Mastadenov.: Human mastadenovirus C, Paramyxov.: Paramyxovirues.

In the approach using the untrimmed RNA-Seq data sets for the Taxonomer tool the program detected several virus sequences of diverse virus species in almost all cell lines which had not been detected before. Regarding the aforementioned BL-70 data set, Taxonomer´s quick analysis (“Binner”) categorized 213,249 reads to the major taxonomic categories human (191,676 reads), bacterial (6,170 reads), and viral (43 reads), as well as 1,410 reads to an “ambiguous” bin (sequences that can be categorized to more than one bin) and 13,950 reads to a so called “unknown” bin (sequences that could not be assigned to one of the other bins) (Fig 1). The reads within a bin were further classified according to different features of the sequences: (1) 119,615 human reads deriving from mRNAs, (2) 5,853 bacterial reads deriving from 16S rRNAs, and (3) 41 viral reads identified by the Protonomer module based on the protein database UniProt. Regarding the viral reads, 27 were assigned to the virus species or genera listed in Table 6. A graphic representation of the distribution of the virus species to the different viral taxa is shown in Fig 1A.

**Table 6:** BL-70 RNA-Seq reads from the Taxonomer virus bin assigned to virus species or genera.

Virus species or genus	Family	No. of reads
Agrotis segetum nucleopolyhedrovirus B	Baculoviridae	1
Phthorimaea operculella granulovirus	Baculoviridae	1
Glypta fumiferanae ichnovirus	Polydnaviridae	2
unclassified Coccolithovirus	Phycodnaviridae	2
Synechococcus phage S-ShM2	Myoviridae	1
Salmonella phage SPN3US	Myoviridae	1
Pectobacterium phage PM1	Myoviridae	1
unclassified T4-like virus	Myoviridae	1
Pandoravirus salinus	unclassified dsDNA viruses	1
	unclassified dsDNA viruses	1
White spot syndrome virus	Nimaviridae	1
Suid herpesvirus 1	Herpesviridae	3
Chelonid herpesvirus 5	Herpesviridae	1
Rat cytomegalovirus Maastricht	Herpesviridae	1
	unclassified dsDNA virus	1
Gammaretrovirus	Retroviridae	2
Bovine viral diarrhea virus 1	Flaviviridae	2
Pestivirus (not assigned to BVDV1)	Flaviviridae	2
Hepatitis C virus	Flaviviridae	1
Strawberry polerovirus 1	Luteoviridae	1
undefined virus reads		14

As shown in S2 Table, between 0 and 59 classified reads were assigned to up to 18 different virus families for the individual non-infected cell lines. To evaluate the virus associated reads, we analyzed the sequences in detail and examined the quality scores of the reads. We chose the cell line MHH-CALL-4 which was shown to reportedly carry HTLV-1 specific sequences by direct alignment (see section on direct comparison of PCR-based virus detection and RNA-Seq data set analysis). As shown in S3 Table, most of the “viral” reads of the cell line MHH-CALL-4 exhibited at least partially a low sequencing quality represented by a significant portion of “#” symbols, which typify a P error of 0.63096. Almost all sequences showed no significant similarity performing a BLASTn analysis and two of the partially low or completely low-quality reads exhibited at least partial similarity to two human genes. On the other hand, three of partially or completely good quality viral reads revealed almost complete homology to XMLV sequences.

The numbers of classified reads assigned to viruses in general and the number of identified virus families they were attributed to are summarized in S2 Table. The table shows that the virus infected cell lines can readily be identified by the highly elevated number of virus specific reads compared to the cell lines shown to be virus negative by PCR. On the other hand, similar numbers of classified virus reads and virus families indicate that only a few reads were assigned to the individual virus families and that they are most likely a result of low-quality reads or contaminations.

To exclude that the poor-quality reads of an RNA-Seq analysis are accumulated in the first part of the data file, we used the full analysis mode of Taxonomer screening 2 x 10^7^ reads. The cell line CADO-ES-1 with 50 reads assigned to 12 different virus families revealed one of the highest read number for virus negative cell lines employing the quick analysis mode for 213,000 reads. The full analysis revealed 4,257 reads assigned to 28 virus families. The vast majority of the reads were assigned to the virus families identified by the quick analysis. This demonstrates that the quick analysis represents well the result of an almost hundredfold number of reads. The number of hits of the quick analysis varies by +10% relative to the full analysis of the CADO-ES-1 cell line. Similar results were obtained for the cell lines DOHH-2 (-6%) and HCC-827 (+12%). The quick analysis of the cell lines HCC-827 and KASUMI-1 showed a 40% and 46% higher number of virus specific reads compared to the full analysis, respectively. On the other hand, the cell lines HCT-15, KASUMI-6, KYSE-30, and MHH-CALL-4 revealed lower virus specific hits of -49%, -38%, -68%, and -43%, respectively, for the quick analysis. Taking into account, that the virus specific hits of those cell lines were in the range between 6 (HCT-15 and KASUMI-6) and 50 (CADO-ES-1), all viruses would have been detected with the full set of reads (100x more reads). We also compared the quick analysis of the EBV positive cell line CI-1 exhibiting an intermediate number of virus specific reads with its full analysis. The quick mode classified 193 reads as viruses whereas the full analysis mode assigned 18,708 reads as viruses. This corresponds to +2.6% with respect to the quick analysis. The results demonstrate that the virus specific reads are largely evenly distributed in the sequencing files.

**Fig 2:**
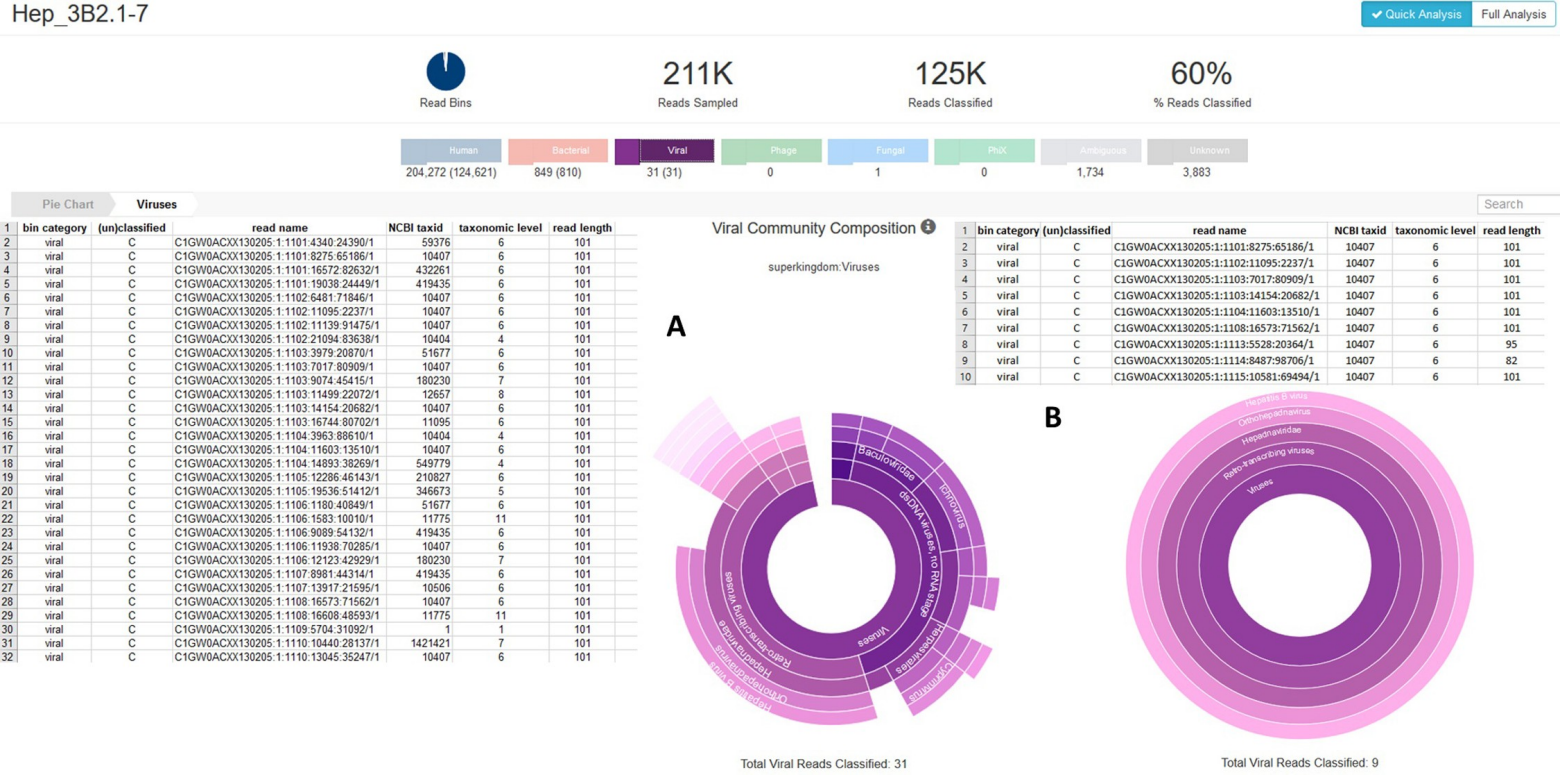
Virus detection in the hepatitis B positive cell line Hep_3B2.1–7 applying the Taxonomer bioinformatics tool before and after trimming. Shown are analysis outputs of quick analyses of 210,769 reads. Of those, 125,462 reads (60%) were classified within the human, bacterial, and viral bins (numbers in brackets). A) The table lists all reads categorized to the viral bin before trimming. The reads with a taxonomic level higher than 1 are depicted in the circular diagram. B) The table lists the remaining viral reads after trimming the reads using the Trimmomatic tool (SLIDINGWINDOW:4:30; MINLEN:80) and the nine consistently classified HBV reads in the circle.

The cell line Hep-3B2.1–7 exhibits similar numbers of virus specific reads and virus families. Taxonomer determined twelve Hepadnaviridae of which ten were assigned to HBV in cell line Hep-3B2.1–7 beside 19 reads of 12 other genera in 211,000 analyzed reads. Trimming the reads applying a sliding window of four with a mean phred score of 30 and a minimal length of 80 bp ended up in nine HBV specific reads and no additional virus specific reads (Fig 2A and 2B). Extrapolating the 12 HBV specific reads to the total number of 71 million reads results in 4,050 HBV specific reads. This number is in the range of what was detected by the direct genome alignment (n = 2,695) and confirms the relatively low transcription rate of HBV genes in comparison to other virus contaminations. As the HBV infection was verified previously by PCR this relatively low number of virus specific reads represents an authentic infection and needs to be demarcated from the other virus detections with similar or even more virus specific read numbers which can be traced back to sample contaminations. But concerning HBV, only 16 further cell lines revealed HBV specific alignments for only one to four reads. This indicates that sample contamination with HBV sequences is unlikely. As shown in Table 2, the same is true for HHV-8, HPV, HIV-1 and -2, HTLV-2 and HCV, whereas EBV, XMLV (incl. XMRV), SMRV and HTLV-1 are prone to sample contaminations.

**Fig 3:**
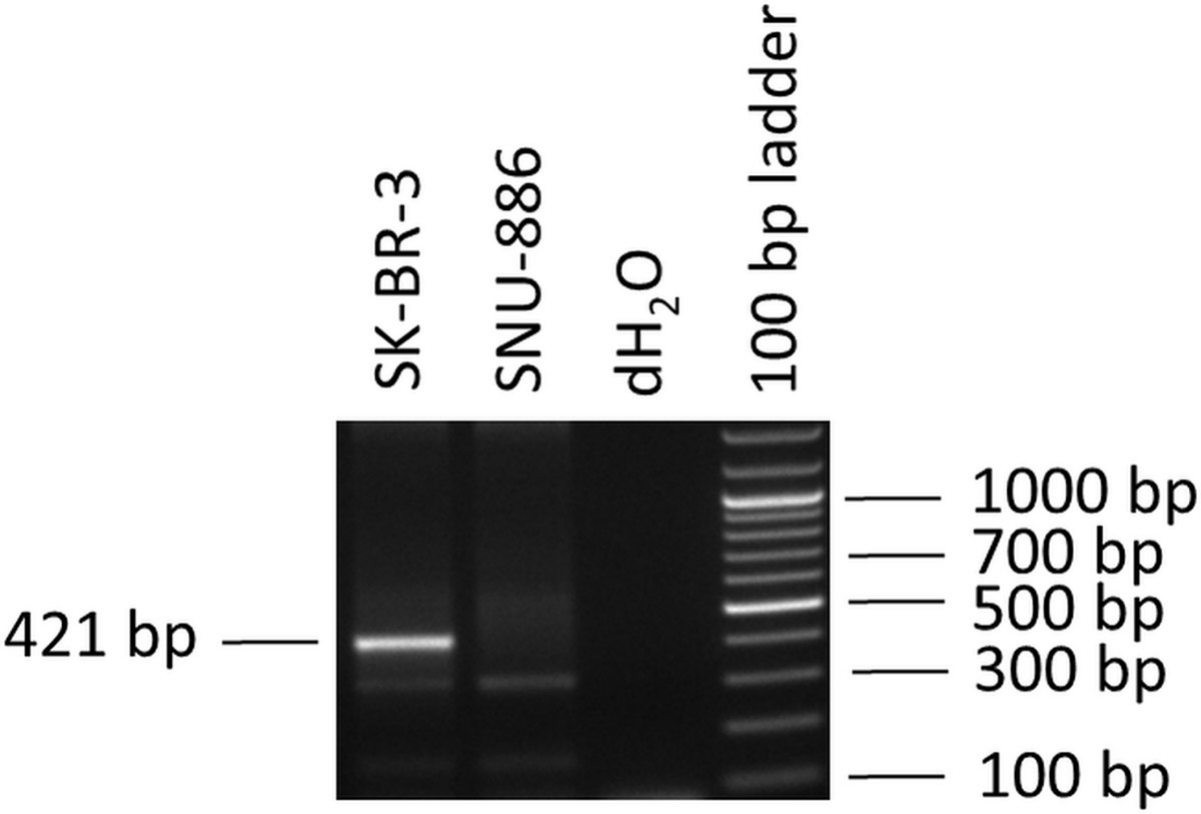
Detection of BPyV by PCR. Shown is an ethidium bromide-stained gel containing the reaction products following PCR amplification with the primers QB-F1-1 and BPyV-rev. A product of 421 bp was obtained. Genomic DNA of the BPyV positive cell line SK-BR-3 and of the BPyV negative human liver cell line SNU-886 were used for the detection. The PCR product of SK-BR-3 was subsequently sequenced and aligned to BPyV sequences of the NCBI nucleotide database showing complete homology.

Concerning the virus infected cell lines, the results agree well with the results of the virus specific determinations of the RNA-seq data as well as the PCR results. During the course of the study we found one virus infection that had not been detected before: the SK-BR-3 revealed 163 reads of BPyV. This infection was verified by a BPyV specific PCR assay (Fig 3).

BPyV was first detected in simian kidney cell lines and later shown to be of bovine origin [29]. The virus was transmitted from the fetal bovine serum to the cells. Due to the close relation of apes and man a transmission from FBS to human cell lines appears to be possible. Schuurman et al. determined an infection rate of more than 70% in different sera and that all PCR-positive sera contained infectious viruses [30]. Like other polyomaviruses (e.g. SV40) BPyV exhibits a large-T antigen which is responsible for the transforming properties of the virus. However, given the high rate of infected serum batches and the capability of the virus to infect bovine and primate cells it remains unclear why only one cell line was found to be positive for the virus. Furthermore, SK-BR-3 was established from breast carcinoma cells and not from kidney cells from which BPyV was usually isolated. Rare modified BPyV might be responsible for the productive, naturally occurring transmission from one host mammal to another. Further RNA-Seq or PCR studies will probably uncover some more infected cell lines.

Another bovine virus frequently detected in FBS (47%) is the bovine viral diarrhea virus (BVDV) [31]. This RNA virus belonging to the Flaviviruses can be detected in many cell lines of cloven-hoofed animal origin (e.g. FLK-BLV, LAT, EBL, MDBK) (determined by RT-PCR, results not shown). Cell lines from diverse animal species and human origin could be infected [32, 33]. But until now, no human cells lines were found to be positive for BVDV.

The results of the present study demonstrate that the methods used agree very well and that RNA-Seq data sets can conveniently be used for the detection of viruses in cell lines. Utmost attention should be paid during NGS laboratory preparation and bioinformatics analysis of RNA-Seq data sets, particularly in regards to: 1) RNA extraction and library construction in designated hoods/areas where proper cleaning procedures are in place to minimize cross-contamination, 2) quality filtering procedures for removal of primers/adapters and low quality bases. The latter point is particularly important for the Taxonomer analysis tool because all sequences are compared to several sequence databases relevant for human samples infected with microbiological agents. This also includes the protein data bases of viruses (UniProt). Taking into account that a six-frame translation is performed for the alignment with protein sequences, the poor-quality sequences may yield a number of false positive results. However, when 200,000 reads are analyzed those reads produce only very few hits per assigned virus family compared to hundreds of reads assigned to a single virus family regarding true positive samples. Nevertheless, filtering poor-quality reads of an RNA-Seq analysis by specific data processing tools (e.g. Trimmomatic) is recommended. Furthermore, dual-matched indexed adapters can be used to eliminate index misassignment when massively parallel sequencing is performed. If the handicaps of contamination and poor-quality reads are considered and carefully handled, all detection methods are well suited for the risk assessment of cells. The Taxonomer results show that the panel of viruses used for characterization of cell lines covers the vast majority of virus contamination. Furthermore, no “new” viruses that were previously not considered to be present in cell cultures could be identified.

## Supporting information

S1 Table Number of WES reads mapped to viral reference sequences. * Cell lines previously shown by PCR to be virus infected. For information on the cell lines and the infections determined by PCR, please refer to S2 Table.
(XLSX)

S2 Table Cell lines used for the Taxonomer analysis. (XLSX)

S3 Table Read sequences assigned by Taxonomer to the viruses bin aligned to NCBI sequences after BLASTn search of the database. (XLSX)
